# Multidisciplinary Atrial Fibrillation Clinics: A Model for Comprehensive Management

**DOI:** 10.31083/RCM38739

**Published:** 2025-09-24

**Authors:** Mahmoud Abdelnabi, Firas Ashour, Cristian Rodriguez, Chanokporn Puchongmart, Ben Thiravetyan, Diego Cruz, Ramzi Ibrahim, Hoang Nhat Pham, Eiad Habib, Christopher Kanaan, Reza Arsanjani, Dan Sorajja

**Affiliations:** ^1^Department of Cardiovascular Medicine, Mayo Clinic, Phoenix, AZ 85054, USA; ^2^Department of Internal Medicine, Texas Tech University Health Sciences Center, Lubbock, TX 79430, USA; ^3^Department of Medicine, University of Arizona, Tucson, AZ 85719, USA

**Keywords:** atrial fibrillation, clinics, cerebrovascular stroke, thromboembolic events, risk factors, multidisciplinary clinics, prevention

## Abstract

Atrial fibrillation (AF) is the most common cardiac arrhythmia and is associated with increased morbidity, mortality, and healthcare expenses. Meanwhile, effective management of AF requires a comprehensive, patient-centered approach that includes risk factor modification, rate and rhythm control strategies, and prevention of thromboembolic events. Multidisciplinary AF clinics have emerged as a model for comprehensive management to improve patient outcomes and reduce healthcare burden. This review discusses the rationale, structure, benefits, challenges, and future directions of multidisciplinary AF clinics, emphasizing their role in optimizing AF management.

## 1. Introduction

### 1.1 Prevalence and Risk

Atrial fibrillation (AF) is the most common sustained arrhythmia worldwide, with an estimated global prevalence of 50 million in 2020. In the United States, the American Heart Association/American College of Cardiology (ACC) indicates that the prevalence of AF was 5.2 million in 2010 and is projected to rise to 12.1 million by 2030, with an expected increase in incidence from 1.2 million to 2.6 million cases per year over the same period. Lifetime risk is approximately 30–40% in White individuals, 20% in African American individuals, and 15% in Chinese individuals [1]. Additionally, the lifetime risk of AF for individuals aged 40 years and older is approximately 26% for men and 23% for women, with men consistently at higher risk across populations [2, 3].

### 1.2 Clinical and Economic Burden

AF is also associated with significant morbidity and mortality. AF is associated with a 1.5–2-fold increased risk of death and significantly elevated risks of heart failure (5-fold), chronic kidney disease (1.6-fold), and peripheral artery disease (1.3-fold). Additionally, AF patients have higher rates of hospitalization and other adverse outcomes, such as cognitive impairment, stroke, myocardial infarction, and sudden cardiac death [1, 3]. AF is associated with a 2.4-fold increased risk of stroke, with the absolute stroke rate averaging approximately 3.5% per year for 70-year-old individuals with AF. The American Heart Association (AHA)/American Stroke Association guidelines indicate that AF increases the risk of ischemic stroke 4- to 5-fold, primarily due to thrombi formation in the left atrial appendage [1, 4]. Overall, AF is associated with significant healthcare costs and resource utilization due to increased inpatient admissions, outpatient visits, and prescription medication costs. According to the ACC/AHA report, annual healthcare costs for individuals with AF are $63,031, which is $27,896 higher than for those without AF. In 2016, AF accounted for an estimated $28.4 billion in US healthcare spending, driven by increased inpatient and emergency department utilization [1]. Nationwide analyses from Europe demonstrate that direct medical costs for AF patients are 50–73% higher than for matched controls, with hospitalizations, especially those related to stroke, being the primary cost driver. In Switzerland, AF-related direct healthcare costs account for approximately 1% of total national health expenditures. Indirect costs, including productivity loss, further increase its societal impact [5, 6].

### 1.3 Multidisciplinary Care of Atrial Fibrillation

Given the substantial multifactorial burden of AF along with its management complexity, which includes symptom control, anticoagulation for thromboembolic event prevention, and comorbidity management, it often requires a multifaceted approach [1]. Multidisciplinary AF clinics are a model that integrates various healthcare professionals to provide patient-centered, coordinated care, addressing the multifaceted needs of AF patients [7]; however, the current guidelines suggest that while a multidisciplinary team approach for managing AF is potentially beneficial, there is insufficient evidence to state that it leads to better outcomes than comprehensive care by a single clinician using an evidence-based clinical care algorithm [8].

## 2. Rationale for Multidisciplinary AF Clinics

### 2.1 Comprehensive Risk Stratification and Management

While AF management often emphasizes acute care, chronic management is frequently underrecognized, highlighting the need for a more comprehensive and sustained approach [9]. A multidisciplinary team approach is utilized to evaluate and manage comorbidities such as hypertension, diabetes, obstructive sleep apnea, chronic obstructive pulmonary disease (COPD), and obesity, which exacerbate and complicate AF. This approach ensures systematic identification and management of risk factors. Hypertension is present in more than 80% of patients with AF. The mechanism of arrhythmogenicity is left ventricular and atrial hypertrophy, along with left atrial enlargement and fibrosis. Adequate control of hypertension, primarily achieved with angiotensin-converting enzyme inhibitors or angiotensin receptor blockers, has been shown to reduce the risk of AF by reducing and reversing cardiac structure and function changes [10]. Diabetes mellitus is not only an independent risk factor for AF, but it also increases symptom burden, increases hospitalization and mortality rates, and reduces quality of life (QoL). Glycemic fluctuations, oxidative stress, and inflammation lead to structural and electrical heart remodeling [11]. Obstructive sleep apnea and AF share many risk factors, including age, sex, obesity, diabetes, and smoking. During periods of apnea, patients experience oxygen and pressure changes that lead to remodeling and fibrosis of the atria. Continuous positive airway pressure use reduces the burden of AF, whether or not patients pursue ablation strategies [12]. Patients with COPD have an increased risk of developing AF due to hypercapnia, hypoxemia, oxidative stress, and increased right heart pressures, which can dilate the right atrium [9, 13]. Obesity increases the risk of AF indirectly, increasing all the other risk factors like diabetes, obstructive sleep apnea, and hypertension, or directly through structural and electrical remodeling. Thus, weight loss has been shown to reduce the risk of new-onset AF and recurrences [14].

### 2.2 Guideline-Directed Care

The ACC/AHA guidelines emphasize the importance of a comprehensive, integrated approach to AF management, highlighting the importance of rhythm and rate control strategies, optimal anticoagulation for thromboembolic prevention, and the management of systemic comorbidities to enhance clinical outcomes and minimize AF-related complications [8]. AF clinics can assist in implementing evidence-based guidelines for lifestyle interventions and rate or rhythm control strategies, such as early catheter ablation referrals and anticoagulation [1]. This evidence-based approach can reduce variations in patient care, reducing morbidity and mortality. Non-cardiologists often face challenges in staying up to date with guideline-directed therapies, particularly when managing patients with complex or multiple comorbid conditions. Contributing factors to suboptimal adherence to AF guidelines include fragmented care delivery, the clinical complexity and heterogeneity of AF, and insufficient patient education [15]. The “Atrial Fibrillation Better Care” pathway addresses three key aspects: stroke prevention, symptom and comorbidities management, and improving patient clinical outcomes. A post hoc analysis of the Atrial Fibrillation Follow-Up Investigation of Rhythm Management (AFFIRM) trial revealed that patients managed according to the Atrial Fibrillation Better Care (ABC) pathway had significantly lower rates of all-cause mortality, composite outcomes of stroke, major bleeding, and cardiovascular death, as well as first hospitalization, compared to those not managed with the ABC pathway [16]. Similarly, the ATHERO-AF study cohort demonstrated that adherence to the ABC pathway was associated with a lower risk of cardiovascular events (CVEs) in a real-world population of patients with non-valvular AF [17]. Moreover, a nationwide cohort study from Korea demonstrated that compliance with the ABC pathway was associated with lower rates of all-cause death and composite outcomes of death, ischemic stroke, major bleeding, and myocardial infarction [18]. Additionally, in a systematic review and meta-analysis, this pathway demonstrated a lower risk of all-cause death (odds ratio of 0.42), cardiovascular death (odds ratio of 0.37), stroke (odds ratio of 0.55), and major bleeding (odds ratio of 0.69) [19]. Based on these findings, the European Society of Cardiology (ESC) guidelines endorsed its role in providing consistent and equitable care across different healthcare settings [20]. Adherence to guideline therapy is complicated, as it requires the physician’s knowledge and coordination with the institution or clinic. For this reason, developing protocols based on the best available evidence in a coordinated clinic setting is necessary to achieve the best possible outcomes [15].

### 2.3 Enhanced Patient Education and Engagement

Patient education on AF risk and management strategies can improve clinical outcomes by supporting mental well-being, improving medication adherence, and minimizing serious adverse events. There is also evidence that patient activation, defined as the state in which patients possess the knowledge, skills, and confidence to manage their healthcare, has been associated with improved health-related quality of life, increased knowledge of the disease, and better medication adherence [21]. Additionally, there is evidence that AF patients’ education is associated with reduced mortality and readmission rates [22]. Comprehensive and coordinated patient-centered education is essential, but limitations exist due to fragmentation in healthcare delivery. This results in inadequate patient education due to time constraints, knowledge gaps, and conflicting information [23].

### 2.4 Improved Coordination of Care

A multidisciplinary approach can improve communication between healthcare teams and providers, facilitate timely cardiology referrals, and minimize treatment initiation delays. This coordinated care model can effectively manage AF, addressing its complexity. Dedicated AF coordinators in specialized clinics can further facilitate this communication, optimizing patient care and overall outcomes [24] (Table 1).

**Table 1.** **Summary of rationale for multidisciplinary AF clinics**.

Domain	Key points	Evidence highlights
Risk stratification & management	Systematic control of comorbidities (HTN, DM, OSA, COPD, obesity)	HTN in >80% of AF patients; CPAP, weight loss reduce AF burden
Guideline-directed care	Consistent use of evidence-based protocols (e.g., ABC pathway)	ABC pathway lowers mortality, stroke, and bleeding rates
Patient education & engagement	Improves adherence, QoL, and self-management	Linked to lower mortality and readmissions
Care coordination	Enhances communication, referrals, and timely treatment	Dedicated clinics improve outcomes

Abbreviations: HTN, Hypertension; DM, diabetes mellitus; OSA, obstructive sleep apnea; COPD, chronic obstructive pulmonary disease; QoL, quality of life; CPAP, continuous positive airway pressure; ABC Pathway, Atrial Fibrillation Better Care Pathway; AF, atrial fibrillation.

## 3. Benefits of Multidisciplinary AF Clinics

### 3.1 Reduction in All-Cause Mortality

AF is independently associated with a significant increase in all-cause mortality, with a 2.5-fold higher risk in women and a 1.5-fold higher risk in men. Despite adherence to guideline-based treatment protocols, sudden death, heart failure, and thromboembolic complications, including stroke, remain frequent contributors to AF-related mortality [1]. A post hoc analysis of a randomized clinical trial demonstrated that integrated specialized AF clinics significantly reduced all-cause mortality compared to usual care. The study showed that patients managed in an AF Clinic had a lower all-cause mortality rate (3.7%) compared to those receiving usual care (8.1%) with a hazard ratio (HR) of 0.44 (95% CI 0.23–0.85; *p* = 0.014) for all-cause mortality and an HR of 0.28 (95% CI 0.09–0.85; *p* = 0.025) for cardiovascular mortality. This reduction in mortality was attributed to both lower cardiovascular and non-cardiovascular deaths [25]. Additionally, the Standard Versus Atrial Fibrillation-Specific Management Strategy (SAFETY) trial found that a disease-specific management program for AF patients was associated with a higher proportion of days alive and out of the hospital. However, it did not significantly prolong event-free survival [26]. Moreover, the ALL-IN cluster randomized trial assessed the effectiveness of integrated care for AF in primary care through quarterly AF check-ups by trained nurses, anticoagulation monitoring, and facilitated consultations with cardiologists. Over a two-year follow-up, the intervention significantly reduced all-cause mortality (3.5 vs. 6.7 per 100 patient-years; HR 0.55, 95% CI 0.37–0.82) and non-cardiovascular mortality (HR 0.47, 95% CI 0.27–0.82), while other adverse events showed no significant differences. These findings highlight the potential of integrated care in reducing mortality among elderly AF patients in primary care settings [27].

### 3.2 Decrease in Acute Hospitalizations

A retrospective cohort study by Frydensberg and Brandes showed that the patients managed within a specialized, multidisciplinary AF clinic experienced a significantly lower incidence of acute hospitalizations compared to those receiving conventional care. Furthermore, a study conducted by Ptaszek* et al*. [28] showed that implementing a multidisciplinary AF management pathway within the emergency department was associated with a significant 5-fold reduction in hospital admission rates and a more than 2-fold decrease in the length of stay for patients requiring hospitalization. These findings suggest that implementing coordinated care pathways can improve clinical outcomes and reduce the burden on hospital resources, thereby potentially enhancing the efficiency of healthcare delivery [24, 28]. Previous data suggest that patients with a higher comorbidity burden had significantly higher rehospitalization rates. Munir* et al*. [29] reported a 30-day readmission rate of 15.1% for patients admitted with AF, with AF and heart failure as the leading causes. Similarly, Tripathi* et al*. [30] found a 14.4% readmission rate, identifying AF as the most common cause and highlighting heart failure, chronic obstructive pulmonary disease, and chronic kidney disease as significant predictors of readmission. A study on the Bridging the Discharge Gap Effectively (BRIDGE) program, a nurse-practitioner-led transitional care clinic, found that program attendance was associated with an 11% lower hazard of hospital readmission and adverse outcomes, particularly in patients with a low Charlson Comorbidity Index (CCI). However, the benefits were significantly less in patients with a high CCI, suggesting that transitional care programs may be less effective in patients with a higher comorbidity burden [31].

### 3.3 Improved Clinical Outcomes

A before-and-after study showed that patients managed in a nurse-run, physician-supervised AF clinic had better composite outcomes, including reduced death, cardiovascular hospitalization, and AF-related emergency department visits, with an odds ratio of 0.71 (95% CI 0.59–1.00; *p* = 0.049) [24]. Evidence has demonstrated that multidisciplinary AF clinics reduce hospitalizations, emergency department visits, and AF-related complications. Patients in these clinics are more likely to receive appropriate anticoagulation and comorbidity optimization and undergo timely rhythm control procedures [32]. Additionally, the ACC/AHA guidelines recommend catheter ablation to improve symptoms and quality of life in symptomatic paroxysmal or persistent AF and as a first-line therapy in selected younger patients with few comorbidities and a moderate to high AF burden. Catheter ablation is also considered for reducing AF progression and associated complications in selected asymptomatic or minimally symptomatic patients [1]. Multidisciplinary AF clinics can optimize patient selection, risk factor management, and pre-and post-procedural care, which are critical for maximizing ablation outcomes and minimizing recurrence. Early rhythm control, including ablation, is associated with improved cardiovascular outcomes and reduced progression to persistent AF, especially when implemented soon after diagnosis [1, 2, 3].

### 3.4 Patient Adherence

Adherence to AF management is significantly affected by the educational backgrounds of both patients and their caregivers [33]. Despite this, data suggests that medication adherence rates remain unacceptably low, with only approximately 20% of patients fully compliant with their prescribed regimens [34, 35]. However, adherence improves significantly with a multidisciplinary approach, such as in lifestyle management for obese patients with AF, achieving a 95% adherence rate in the first year [36].

### 3.5 Patient Satisfaction

Evidence suggests that specialized AF clinics adopting multidisciplinary models can significantly enhance patient satisfaction and compliance. The NICE-AF clinic, a nurse-led integrated chronic care model, significantly improved patient satisfaction (*p* = 0.020) and AF-specific QoL, patient knowledge of AF, and medication adherence [34, 37]. Patient-reported outcome measures (PROMs) are increasingly recognized as an essential tool for AF management, providing insights into patient symptoms and QoL to evaluate treatment strategies. A systematic review by Kotecha* et al*. [38] identified several AF-specific health-related quality-of-life questionnaires, including the Atrial Fibrillation Effect on QualiTy-of-Life (AFEQT), demonstrating good reliability and validity, making it a reliable tool for clinical use. The Utah mEVAL AF Program demonstrated that higher AF symptom severity scores, as measured by the Toronto AF Symptom Severity Scale (AFSS), were associated with more aggressive clinical management, including increased rhythm control strategies [39]. Additionally, the AHA recommendations focus on the importance of measuring patient-reported health status in cardiovascular health, including AF, to enhance patient-centered care and improve clinical outcomes [40].

### 3.6 Psychosocial Management

Emerging evidence indicates that psychological conditions such as anxiety and depression are common in AF patients, affecting approximately 28–40% of this population, and play a significant role in adherence, QoL, and even prognosis [41]. The relationship between AF and psychological distress is bidirectional: AF symptoms can precipitate anxiety and depression, while psychological distress increases symptom burden, healthcare utilization, and may worsen clinical outcomes [41, 42]. Routine screening for depression and anxiety using validated tools such as the Patient Health Questionnaire-9 (PHQ-9) and Generalized Anxiety Disorder-7 (GAD-7) is recommended for screening per ACC/AHA guidelines to identify patients at risk for poor adherence and outcomes, facilitating timely intervention [1, 43]. Cognitive behavioral therapy (CBT) and mindfulness-based interventions have demonstrated efficacy in improving psychological well-being, coping skills, and QoL in AF patients [44, 45]. Nurse-led behavioral activation programs and structured patient education interventions also reduce anxiety and depression, improve medication adherence, and empower patients in shared decision-making, with sustained benefits at six months [21]. Catheter ablation, when effective in reducing AF burden, is associated with significant and sustained reductions in psychological distress and suicidal ideation compared to medical therapy alone, suggesting that rhythm control may directly alleviate psychological symptoms in select patients [42]. Stress management strategies may reduce AF episodes and improve psychological outcomes, though further evidence is needed to establish their role [46].

### 3.7 Value-Based Care Considerations for Multidisciplinary AF Clinics

While integrated care models have many benefits in theory, their implementation in daily clinical practice remains challenging. Significant barriers include time limitations, insufficient economic resources, the necessity for improved collaboration among primary, auxiliary, and tertiary care domains, and shortcomings in postgraduate education for healthcare practitioners [34, 47].

### 3.8 Healthcare System Fragmentation

The US healthcare system is significantly fragmented, resulting in patients receiving healthcare from several providers. In 2019, 35% of Medicare beneficiaries consulted five or more physicians. Despite the prevalence of this fragmentation, limited efforts have been made to address the issue so far [48]. This fragmentation results in considerable limitations in delivering effective and coordinated care. Successful multidisciplinary care can facilitate collaboration among specialists, which is often affected by various organizational and institutional challenges [35].

### 3.9 Resource Availability

Specialized resources are essential to effectively implementing multidisciplinary care models. Previous studies consistently showed the benefits of nurse-led protocols in multidisciplinary clinics [33], while some tertiary centers include electrophysiologists supervising the care team [7]. Discrepancies in resource availability between academic institutions and non-teaching hospitals can hinder the efficacy of these care models. For example, academic centers typically possess a broader range of resources that may be lacking in smaller or non-teaching facilities [35]. The nurse-led multidisciplinary clinics for AF have significantly reduced emergency room visits for patients with AF [49] and have decreased the necessity for cardiology referrals [50]. However, a clear comparison between nurse-led and physician-led care models at different hospital levels has not been evaluated.

### 3.10 Cost-Effectiveness

An analysis in a Canadian setting found that multidisciplinary AF clinics were cost-effective, providing cost savings and improved quality-adjusted life years (QALYs) compared to usual care [32]. A study published in Europace by the European Society of Cardiology compared a nurse-led integrated chronic care model to standard care, suggesting that a nurse-led intervention resulted in an incremental gain of 0.009 quality-adjusted life years and reduced costs equal to €1109 per patient. Moreover, the nurse-led program demonstrated a higher likelihood of being cost-effective compared to usual care across all plausible thresholds of willingness to pay for quality-adjusted life years, suggesting its economic viability in managing chronic conditions [51]. Additionally, a study from the University of Pittsburgh demonstrated that directing patients with AF to a dedicated center significantly reduced overall costs, primarily by lowering emergency department care costs, although outpatient costs were slightly higher. The average cost at 30 days was $619 compared to $1252 for usual care, with these differences persisting at 90 days [52].

### 3.11 Innovation and Research

AF clinics can serve as platforms for clinical research by facilitating the systematic collection of real-world data, which is essential for understanding clinical outcomes and the effects of AF management strategies in diverse patient populations. Large data registries such as the HERA-FIB registry, which enrolled over 10,000 patients, provide significant insights into AF management in a real-world setting, including data about underrepresented populations in clinical trials. These registries are essential for bridging the gap between clinical trials and daily clinical practice by validating evidence and enhancing the generalizability of guideline-recommended, evidence-based management [53] (Table 2).

**Table 2.** **Summary of key benefits and supporting evidence of multidisciplinary AF clinics**.

Domain	Key findings and benefits	Supporting studies/comments
All-cause mortality reduction	Significant reduction in all-cause and cardiovascular mortality rates	AF Clinic HR 0.44; ALL-IN trial HR 0.55; SAFETY trial increased days alive and out of hospital
Hospitalizations	Lower acute hospitalization and readmission rates	5-fold lower admission (Ptaszek); 11% reduced hazard of readmission (BRIDGE); lower ED visits (nurse-led care)
Clinical outcomes	Better composite outcomes: fewer deaths, CV hospitalizations, ED visits; optimized rhythm control and ablation readiness	OR 0.71; early rhythm control linked to better outcomes
Patient adherence	Improved medication and lifestyle adherence with multidisciplinary teams	95% adherence in lifestyle programs; baseline adherence only ∼20%
Patient satisfaction & QoL	Higher satisfaction and QoL scores, better patient knowledge and PROMs	NICE-AF clinic improved satisfaction; AFEQT and AFSS tools validated
Psychosocial health	Improved anxiety, depression, and stress outcomes via screening and behavioral interventions	PHQ-9/GAD-7 screening, CBT, mindfulness, nurse-led programs
Value-based care	Barriers include lack of time, resources, inter-provider coordination, and training	Calls for improved integration across care levels and better education
System fragmentation	Multidisciplinary care mitigates fragmentation through better coordination among multiple providers	35% of medicare patients saw ≥5 providers annually; limited systemic strategies to address this
Resource availability	Variability in care models and success tied to available staff (nurses, EPs); differences between academic and community centers	Nurse-led models reduce ED visits and cardiology referrals
Cost-effectiveness	Demonstrated savings with increased QALYs; lower overall and ED costs despite higher outpatient expenses	Canadian and European studies; 30-day costs $619 vs. $1252 (U. Pittsburgh)
Research and innovation	AF clinics enable real-world data collection and inclusion of underrepresented groups for better evidence translation	HERA-FIB registry (>10,000 pts); supports external validity of guidelines

Abbreviations: AF, atrial fibrillation; HR, hazard ratio; OR, odds ratio; CV, cardiovascular; QoL, quality of life; PROMs, patient-reported outcome measures; ED, emergency department; PHQ-9, Patient Health Questionnaire-9; GAD-7, Generalized Anxiety Disorder-7; CBT, cognitive behavioral therapy; NICE-AF, nurse-led integrated chronic care for elderly with AF; AFEQT, Atrial Fibrillation Effect on Quality-of-Life Questionnaire; AFSS, Atrial Fibrillation Symptom Score; QALYs, quality-adjusted life years; Eps, electrophysiologists; HERA-FIB, Health Economics and Real-world Assessment in Atrial Fibrillation Registry.

## 4. Future Directions

The future directions of applying multidisciplinary AF clinics are focused on enhancing patient outcomes and streamlining care. First, integrating chronic care models, including multidisciplinary team care, nurse-led AF clinics, and telemedicine, is expected to improve AF-related outcomes by addressing current gaps in care, such as suboptimal anticoagulation, symptom management, and comorbidity control [47]. Second, the Society of Thoracic Surgeons emphasizes the importance of multidisciplinary collaboration between cardiothoracic surgeons and electrophysiologists to enhance patient outcomes post-surgical ablation, suggesting regular monitoring and long-term follow-up [54]. Third, the European Heart Rhythm Association (EHRA) and the Atrial Fibrillation NETwork (AFNET) highlight the potential of artificial intelligence (AI) to support AF screening, rhythm management, and the identification of atrial cardiomyopathy and cognitive impairment, which requires advanced interdisciplinary collaboration [55]. The ACC also recommends a comprehensive care approach tailored to individual patient needs, including lifestyle and risk factor modification and clinician coordination to improve outcomes [8]. Lastly, there is a call for the inclusion of psychosocial management into holistic, integrated care for AF patients, addressing psychological challenges that impact treatment adherence and overall quality of life [34].

### 4.1 Telemedicine 

Telemedicine and mobile health units can increase the accessibility and availability of multidisciplinary AF clinics, particularly in rural and underserved areas. The ESC has implemented the TeleCheck-AF approach, which integrates app-based heart rate and rhythm monitoring with structured teleconsultations to ensure comprehensive AF management [56]. This approach can be utilized for continuous remote monitoring and management of patients in rural and underserved areas. mHealth solutions, such as the TeleCheck-AF and TeleWAS-AF projects, can facilitate on-demand monitoring and support a wait-and-see strategy for recent-onset AF, reducing the burden on emergency department visits and enhancing patient involvement in their care [57, 58]. Additionally, telemedicine has been shown to improve clinical outcomes through early detection and timely interventions for AF episodes. For instance, trans-telephonic ECG monitoring has demonstrated effectiveness in diagnosing symptomatic paroxysmal cardiac arrhythmias, including AF. It has influenced treatment decisions such as ablation and antiarrhythmic therapy [59].

### 4.2 Integration of AI 

AI introduction into AF management can enhance risk stratification, decision-making, and predictive analytics, ultimately improving patient outcomes. AI can integrate data from several datasets, including electronic medical records (EMR) [60], standard 12-lead ECG [61], and photoplethysmography data from wearable devices [62, 63]. In a prospective trial, AI showed the potential to identify high-risk patients for targeted AF screening [64]. Additionally, AI showed promising results in identifying AF patients, even in sinus rhythm [61], which can detect previously undiagnosed paroxysmal AF early. Moreover, AI models can predict adverse outcomes such as stroke risk [65] or the success of cardioversion [66]. Despite these advances, prospective research is required to validate the real-world applicability and generalizability [67]. Furthermore, concerns regarding AI use in healthcare, such as safety, accuracy, data integrity, and physician oversight, should be addressed before widespread adoption [68].

### 4.3 Standardization of Care Models

Standardized, evidence-based best practices are essential for consistency in multidisciplinary AF clinics. This healthcare model has been associated with improved patient outcomes, including reduced hospitalizations, emergency room visits, cardiovascular mortality, and reduced healthcare costs [49, 69, 70]. In addition, efficiency improvement, as evident by the faster diagnosis-to-ablation time, has been observed with a structured approach [71]. A standardized workflow can enhance efficiency, allowing providers more time for personalized, patient-centered care [72]. However, standardized practice must be balanced to address each patient’s individualized risk factors and social determinants of health.

### 4.4 Focus on AF Prevention

Broadening the scope of AF clinics to include primary prevention strategies and early intervention in high-risk populations is crucial for reducing the disease burden. In addition to traditional risk factors, genetic predisposition, health behaviors, and social factors have been associated with AF with population-attributable risks of 14.3–19.1%, 8.7–11.5%, and 5.5–6%, respectively [73]. A multidisciplinary AF clinic provides an ideal setting to address these factors and implement targeted preventive strategies, which could ultimately contribute to more effective AF prevention and better long-term outcomes (Table 3).

**Table 3.** **Summary of future directions of multidisciplinary AF clinics**.

Theme	Key elements	Anticipated impact
1. Chronic care integration	- Nurse-led clinics	Improved symptom control, anticoagulation adherence, and comorbidity management
	- Telemedicine- Comorbidity and anticoagulation management	
2. Telemedicine	- TeleCheck-AF, TeleWAS-AF projects	Increased access in underserved areas; reduced ED visits; early diagnosis and intervention
	- App-based HR monitoring	
	- Remote consultations	
3. AI integration	- Use of EMR, ECG, and wearable data	Enhanced risk stratification, early detection, and decision support; need for validation and safeguards
	- Risk prediction models	
	- Screening in sinus rhythm	
4. Standardization of care	- Evidence-based workflows	Reduced hospitalizations and costs; more efficient and consistent care delivery
	- Timely diagnosis-to-ablation	
	- Personalization balance	
5. AF prevention	- Addressing genetic, behavioral, and social determinants	Reduced AF incidence; improved long-term outcomes through targeted primary prevention
	- High-risk screening	
6. Psychosocial integration	- Comprehensive care, including mental health support	Improved adherence, quality of life, and patient engagement

Abbreviations: AF, atrial fibrillation; HR, heart rate; ED, emergency department; AI, artificial intelligence; EMR, electronic medical record; ECG, electrocardiogram; mHealth, mobile health.

## 5. Conclusion

Multidisciplinary AF clinics can offer a comprehensive, patient-centered approach to managing atrial fibrillation by integrating specialists, optimizing control of risk factors, adhering to guideline-directed therapy, and providing patient education. These clinics have demonstrated lower mortality, reduced hospitalizations, improved treatment adherence, and cost-effectiveness compared to standard care. Despite challenges in implementation, tools such as standardized protocols, telemedicine, and AI tools can enhance accessibility and efficiency. Expanding preventive strategies and interdisciplinary collaboration can improve patient outcomes and reduce healthcare burden.
